# Graphical tools for macromolecular crystallography in *PHENIX*

**DOI:** 10.1107/S0021889812017293

**Published:** 2012-05-16

**Authors:** Nathaniel Echols, Ralf W. Grosse-Kunstleve, Pavel V. Afonine, Gábor Bunkóczi, Vincent B. Chen, Jeffrey J. Headd, Airlie J. McCoy, Nigel W. Moriarty, Randy J. Read, David C. Richardson, Jane S. Richardson, Thomas C. Terwilliger, Paul D. Adams

**Affiliations:** a Lawrence Berkeley National Laboratory, One Cyclotron Road, Mailstop 64R0246, Berkeley, CA 94720, USA; b Department of Haematology, University of Cambridge, Cambridge Institute for Medical Research, Wellcome Trust/MRC Building, Cambridge CB2 0XY, UK; c Department of Biochemistry, Duke University Medical Center, Durham, NC 27710, USA; d Los Alamos National Laboratory, Los Alamos, NM 87545, USA; e Department of Bioengineering, UC Berkeley, CA 94720, USA

**Keywords:** macromolecular crystallography, graphical user interfaces, *PHENIX*

## Abstract

A new Python-based graphical user interface for the *PHENIX* suite of crystallography software is described. This interface unifies the command-line programs and their graphical displays, simplifying the development of new interfaces and avoiding duplication of function. With careful design, graphical interfaces can be displayed automatically, instead of being manually constructed. The resulting package is easily maintained and extended as new programs are added or modified.

## Abstract 2

The foundations and current features of a widely used graphical user interface for macromolecular crystallography are described.

## 1. Introduction

*PHENIX* (Adams *et al.*, 2010 ▶) is a widely used system that has been developed for crystallographic structure determination using diffraction data. Recent improvements in the experimental and computational tools available for macromolecular crystallography have led to widespread adoption of the technique by nonspecialists over the past decade, as well as a steep increase in the number of structures deposited in the Protein Data Bank (PDB; Bernstein *et al.*, 1977 ▶; Berman *et al.*, 2000 ▶; Berman, 2008 ▶). Simultaneous efforts by structural genomics initiatives (see for example Cymborowski *et al.*, 2010 ▶; Elsliger *et al.*, 2010 ▶) and the biotechnology and pharmaceutical industries have focused on high-throughput technologies, with the goal of rapid structure solution. Rapid structure solution often requires a combination of many approaches, such as parallel testing of phasing strategies or quick evaluation of multiple related data sets of unknown quality. This can be accomplished by using a high degree of automation, both increasing the probability of successfully solving and refining structures, and avoiding burdening the crystallographer with repetitive time-consuming tasks. A large number of systems for running part or all of the computational ‘pipeline’ nearly unattended have been proposed, many focused around linking together existing components with command-line scripts or HTML interfaces (Brunzelle *et al.*, 2003 ▶; Holton & Alber, 2004 ▶; Kroemer *et al.*, 2004 ▶; Panjikar *et al.*, 2005 ▶; Vonrhein *et al.*, 2007 ▶). In favorable cases, a mostly complete model can be obtained starting from raw diffraction images or processed data; however, additional correction and completion by experts is almost always necessary, and automation may fail for more difficult structures.

In many respects a graphical user interface (GUI) is highly suitable as an automation platform, especially for novice users who face a steep learning curve when using software that implements a large number of complex algorithms. Presentation in a GUI can be used to separate commonly used parameters from advanced options and provide additional information about their intended use. Additionally, a GUI can provide a framework for tracking information about individual projects, rather than relying on the native file system and text files; it can also facilitate automation, for example by suggesting or easing transitions between programs, or promoting re-use of common parameter sets and other input files. Finally, much of the information generated by crystallography software can be difficult to interpret when presented as plain text files. In many cases presentation as two-dimensional graphs or three-dimensional models and maps greatly simplifies the process.

Most of the current generation of graphical interfaces have focused on making the individual steps as accessible as possible and linking them together, rather than imposing ‘black-box’ automation on the user (Potterton *et al.*, 2003 ▶; Pape & Schneider, 2004 ▶; Minor *et al.*, 2006 ▶; Emsley *et al.*, 2010 ▶). We have implemented a graphical interface to *PHENIX* designed around a similar concept, using a novel parameter syntax suitable as a basis for both command-line tools and the GUI. The new GUI provides access to nearly all of the command-line features of *PHENIX*, making it suitable for experts as well as users with a less technical background.

## 2. Methods

### 2.1. The *PHENIX* software development environment

#### 2.1.1. Programming languages

When building a complex large-scale system the choice of fundamental tools is critical. Clearly the most important choice of all is the choice of programming languages. For *PHENIX* a combination of Python (Van Rossum & Drake, 2003 ▶) and C++ (Stroustrup, 2000 ▶) is used (Grosse-Kunstleve *et al.*, 2002 ▶; Adams *et al.*, 2010 ▶). This decision was motivated by a number of considerations. Firstly, Python and C++ are simultaneously compatible and complementary: compatible because both languages are object oriented, complementary because Python maximizes programmer productivity while C++ maximizes runtime efficiency. Secondly, both languages are in wide use in the software development world, including many open-source projects. Therefore this makes accessible a vast and still growing pool of widely used and well maintained libraries. Thirdly, both languages are highly portable and are almost certain to remain so in the future because of the strong community interest in supporting new platforms for the many existing packages based on Python and C++.

The integration between Python and C++ uses the Boost.Python library (Abrahams & Grosse-Kunstleve, 2003 ▶) to implement Python interfaces for the C++ components of *PHENIX*. Boost.Python uses the C++ template engine to generate C++/Python bindings, which has the two important advantages that no additional tools are needed and that the syntax for defining the bindings is standard C++. This contrasts with other widely used tools (most notably SWIG; http://swig.org) thereby maximizing portability and sparing new methods developers the effort of learning a third syntax.

#### 2.1.2. The GUI toolkit

The second fundamental decision is the choice of GUI toolkit. We determined that a Python interface is highly suitable, for two main reasons. The first is that Python maximizes programmer productivity, the second that the core algorithms of *PHENIX* are almost universally called through Python interfaces (Grosse-Kunstleve *et al.*, 2002 ▶). We also determined that the GUI toolkit must offer cross-platform support for all major features and that it should be backed by a major existing user community, because this is correlated with long-term availability. Finally, to be compatible with the distribution model of *PHENIX*, it is important that the GUI toolkit is open source. After a thorough evaluation the wxPython toolkit (http://wxpython.org) was chosen as the best match for these requirements.

#### 2.1.3. A unifying user interface toolkit (Phil)

The third fundamental component behind the *PHENIX* GUI is the Python-based hierarchical interchange language (Phil; http://cctbx.sourceforge.net/libtbx_phil.html), which was developed as part of the *PHENIX* project (Grosse-Kunstleve *et al.*, 2005 ▶). Phil is designed to simultaneously enable intuitive command-line user interfaces and aid in largely automating the generation of graphical user interfaces. *PHENIX* command-line users are presented with a minimal but powerful syntax for defining input parameters; the only two major syntax elements are the equal sign for keyword-value assignments and curly braces for the delineation of a hierarchical structure. For methods developers, Phil provides flexible means for the modular assembly of parameter files. The Phil modules are typically implemented along with the algorithms using the parameters. Phil supports embedded help text for users. Typically the algorithms and the Phil modules including the help text are kept in the same Python source code file. This arrangement ensures that the algorithms, associated parameters and help texts can easily be developed and maintained together and are approachable by new methods developers.

**Figure 1:**
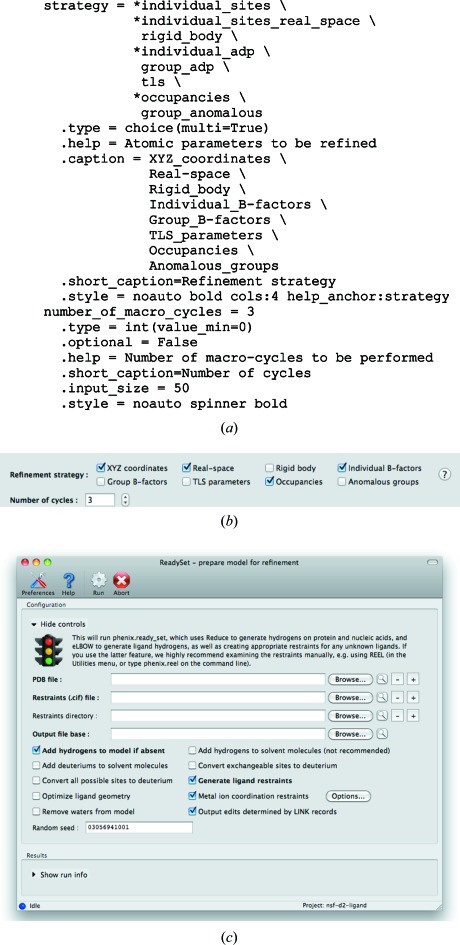
(*a*) Example of *phenix.refine* parameter specifications using the *libtbx.phil* module in *cctbx*, with metadata used in the GUI. (*b*) Controls drawn automatically based on the specifications in (*a*). (*c*) A practical example of fully automatic interface generation: PDB file preparation for refinement in *phenix.ready_set*.

Phil parameter files can be automatically converted to a graphical presentation. This makes use of metadata embedded in the Phil modules, primarily the type for each parameter (for example int, float, choice, path *etc.*), which is defined by the developer. For each type there is a corresponding default presentation in the GUI. Optionally, the type information is augmented by secondary information defined by the developer, to direct customized presentation in the GUI. A typical example is shown in Figs. 1 ▶(*a*) and 1 ▶(*b*). Nearly all of the information required to create and manage the graphical controls for editing these parameters is contained in the metadata. The hierarchical organization of the Phil parameters helps determine the window layout. Developers may modify most of the options for underlying programs without disrupting the GUI. The user-modified parameters are retrieved from the GUI by forming new internal Phil objects, which may be saved as input files for the command-line programs or used directly to start computational processes. Advanced users may opt to edit the Phil files prior to executing the processes. The need for repetitive input is reduced both by the ability to restore and merge past inputs and by the option of saving default settings for specific contexts.

Each Phil parameter definition or hierarchical level is tagged with an ‘expert level’ attribute, which allows the display in the GUI to be limited to basic settings or increasing levels of complexity. Because many of the settings are distracting for novices or used primarily for development purposes, the default interface is kept simple, and a minimal set of controls is displayed in the main window.

The Phil implementation is intended to be reusable for a wide variety of purposes. To this end, the Phil type system is designed to be extensible from Python. For example, the extension mechanism is used to support crystallography-specific types, most notably unit-cell and space-group types. These accommodate common abbreviations and syntactical variations. For example, the space-group symbols ‘p212121’ or ‘19’ are converted to the standardized form ‘P 21 21 21’.

### 2.2. Execution of programs

The *PHENIX* software development framework allows any program to be run within the GUI as a Python module. This enables immediate feedback, such as real-time plots or progress bars. For maximum flexibility, however, several additional approaches to starting processes have been implemented. Programs may be started either directly from the GUI, as separate processes on the same computer, or on a cluster managed by a batch queuing system (for example the Sun Grid Engine or the Portable Batch System). The latter two mechanisms allow the GUI to be closed (and resumed later) without interrupting running processes, at the cost of losing some interactive features that require direct communication between the GUI and running process. In all cases, the printed console output is automatically propagated to the GUI. More complex output is sent to the GUI on a case-by-case basis using ‘callbacks’, which encapsulate intermediate data to be saved to temporary files or sent directly *via* interprocess-communication objects. Information sent to the GUI by this mechanism may include warning messages, tables of statistics, plot data, or even models and maps. The ability to propagate higher-level data to the GUI makes it possible to provide fine-grained indicators of program progress using easily interpretable presentations instead of relying on the text output alone.

### 2.3. Testing

An essential feature of collaborative development, or any long-term software project, is frequent testing of all basic functionality to guard against bugs introduced by code changes. In the context of *PHENIX* and the *cctbx* (Adams *et al.*, 2010 ▶), this is primarily accomplished by running nightly builds and a set of regression tests on all supported platforms. Automated testing of graphical code is notoriously difficult because of the interactive nature of the software. However, careful separation of core logic and presentation allows the nongraphical components to be tested as part of a nightly build system. This includes consistency checks to ensure that none of the parameters represented in the GUI have been removed from the underlying program. Graphical controls are implemented as separate modules with minimal dependencies where possible, which makes them potentially reusable for other purposes and less prone to errors as a result of changes to other code. Each control includes a simple test program that can be run manually as a standalone command, allowing quick verification of code changes without the burden of running the entire application.

## 3. Results and discussion

### 3.1. Overview of the *PHENIX* GUI

As of the current release (1.7.3, December 2011), most of the major programs in *PHENIX* are available in the GUI, including *phenix.refine* (Afonine *et al.*, 2012 ▶), the *AutoSol*, *AutoBuild*, *AutoMR* and *LigandFit* wizards (Terwilliger *et al.*, 2006 ▶, 2008 ▶, 2009 ▶), *Phaser* (McCoy *et al.*, 2007 ▶), *Xtriage* (Zwart *et al.*, 2005 ▶), *eLBOW* (Moriarty *et al.*, 2009 ▶), *MR-Rosetta* (DiMaio *et al.*, 2011 ▶), a comprehensive validation suite largely derived from *MolProbity* (Chen *et al.*, 2010 ▶), *POLYGON* (Urzhumtseva *et al.*, 2009 ▶), and *phenix.model_vs_data* (Afonine *et al.*, 2010 ▶), as well as many simpler programs for building, map calculations, restraints editing, file manipulation, data analysis and visualization.

Nearly all of the programs in *PHENIX* are now configurable by Phil; in those cases where the original code was designed around this framework (such as *phenix.refine* and *Xtriage*), few modifications other than addition of GUI-specific metadata were required. For applications that pre-date Phil, such as the wizards and *eLBOW*, additional wrapper code was required to provide an interface between Phil and the configuration mechanism used internally. In the case of *Phaser*, an entirely new command-line interface was added as a bridge between the Phil layer and the existing Python API (McCoy *et al.*, 2007 ▶).

The Phil-based system has proved essential for maintaining the interfaces to applications such as *phenix.refine*, which currently has more than 900 unique parameters. Most changes to parameters can automatically be propagated to the *PHENIX* GUI, and most customizations are made in the Phil metadata rather than Python code. For particularly complicated input types (especially file input), embedded keywords may specify callback functions to be executed when a parameter is changed. Some common parameters that use the basic types often have additional restrictions on syntax and may be tightly coupled to other parameters, such as the selection of column labels (as comma-separated strings) from reflection data files; these are flagged by style keywords in the Phil syntax and handled by specialized controls. Additional customization of individual applications is often inevitable, and nearly all interfaces contain some custom layout code for the top-level controls. However, for simple programs an entirely automatic interface is possible (Fig. 1 ▶ *c*), and even the most complex interface, that for *phenix.refine*, presents more than 90% of parameters without any customization.

**Figure 2:**
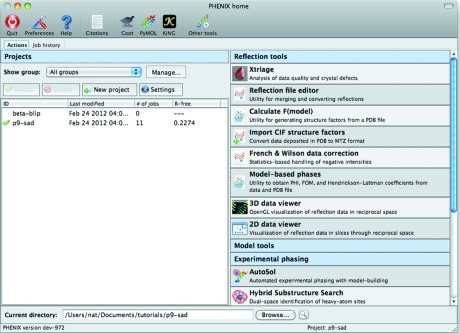
Screen capture of the main window of the *PHENIX* GUI running on a Macintosh computer.

The organization of the central GUI (the ‘phenix’ command) combines a list of user-defined projects and their current status with individual programs grouped by category (Fig. 2 ▶). Internally, Phil is also used to define much of the information about available programs, such as module locations and the content displayed in the widgets in the main GUI. On the Macintosh platform, help links embedded in the Phil specification are used to generate buttons to navigate directly to the HTML documentation from anywhere in the GUI. Phil is also the primary storage format for project tracking. Job history for the currently selected project is displayed in a separate window (reached through the ‘Job history’ tab). Both jobs and projects can be sorted on the basis of modification time or current best *R*_free_ (Brünger, 1992 ▶) (extracted at the end of each job if it is calculated). Currently job tracking is the primary function of projects, but this functionality will be extended in the future to manage default settings and inputs. For demonstration purposes, *PHENIX* is distributed with a number of tutorial data sets, which can be readily set up as projects directly from the GUI.

Although they share a common configuration system, the output of the individual programs is heterogeneous, and a variety of mechanisms have been implemented to present the progress and results of each application visually. Larger programs such as *phenix.refine* send entire molecules and maps *via* callbacks as they are updated, and this output is displayed in graphics programs such as *Coot* (Emsley *et al.*, 2010 ▶) (see below). Final results may encompass validation reports, models, tables for plotting and any information written to files. These results (and any intermediate results from callbacks) are saved to disk and can be quickly reloaded and displayed in the GUI in future sessions. For many programs, the final result returned is a simple list of files created, which can be automatically displayed using a generic procedure. The heterogeneous nature of the large programs and the complexity of the information returned requires the use of manual layout for many results, although re-use of common classes is made wherever possible.

### 3.2. Interfacing with molecular graphics

The *PHENIX* GUI includes basic three-dimensional graphics functionality, for example the ability to display wireframe atomic models and electron density maps. However, manual model building or publication-quality graphics are outside the scope of the *PHENIX* project. Therefore, we have implemented interface layers for the crystallographic building program *Coot* and the general-purpose molecular graphics program *PyMOL* (DeLano, 2002 ▶). Both programs are extensible from Python. Most of the communication is through files [PDB, MTZ or *CCP4* (Collaborative Computational Project, Number 4, 1994 ▶)], but a local network connection is used internally, allowing functions in the *Coot* and *PyMOL* Python APIs (for instance, loading and contouring a map file) to be called remotely from within *PHENIX*. Interactive model validation summaries (‘kinemages’) may also be loaded in *KiNG* (Chen *et al.*, 2009 ▶), providing visual validation similar to that available in *MolProbity*.

All applications in the GUI support the loading of data files directly into *Coot*, and the majority also support *PyMOL* and/or the built-in graphical viewer. Any necessary file conversions or additional commands, such as a fast Fourier transform to generate a *CCP4*-formatted map file, are handled internally. Thus, the final model and map coefficients from refinement can be opened in *Coot* or *PyMOL* by a simple mouse click in the *PHENIX* GUI, and PDB files used for input may be opened in one of the viewers instead of a text editor. A more powerful application of the graphics extensions is continuous display of results during computation, by saving intermediate data and re-loading the files as they change. Similar interactivity has previously been implemented in *VMD* (visual molecular dynamics; Humphrey *et al.*, 1996 ▶), both for visualizing molecular dynamics simulations and for NMR refinement (Schwieters & Clore, 2001 ▶), and applied to visualizing the progress of the building program *ARP*/*wARP* (Payne *et al.*, 2005 ▶; Langer *et al.*, 2008 ▶). This is most useful in refinement where one can watch model and map changes in real time. Other programs that iteratively rebuild models (currently *AutoSol* and *AutoBuild*) produce similar output at longer intervals.

**Figure 3:**
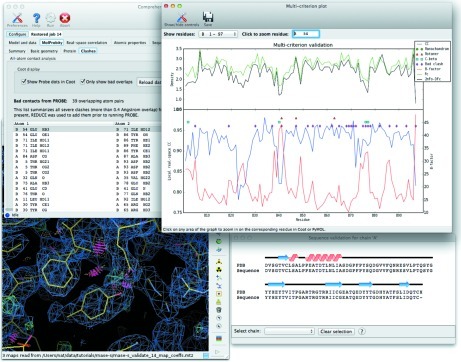
Integrated validation application, showing a list of outliers for all-atom contact analysis, and the multi-criterion plot combining geometry outliers, electron-density levels and *B* factors. The model, maps and Probe dots (Word *et al.*, 1999 ▶) are automatically loaded into *Coot*, and the view is re-centered on the selected atom.

For applications requiring a particularly high degree of interactivity and two-way communication, extending the *Coot* GUI *via* the *Coot* Python API is necessary. This approach has been used to facilitate editing of input models for molecular replacement with the *Sculptor* utility (Bunkóczi & Read, 2011 ▶). In most situations, however, it has been sufficient to use a direct network connection instead. When displaying validation results, therefore, instead of creating a redundant interface in *Coot* to list outliers, the list controls in *PHENIX* re-center the attached *Coot* window on the selected residue (Fig. 3 ▶). Controlling *PHENIX* from *Coot via* the same mechanism is also possible. Currently this is limited to starting the *phenix.refine* GUI and loading a selected model, but more complex operations such as atom picking or selecting from a choice of automatically fitted loops could be added.

### 3.3. Automation in a GUI framework

Although the new *PHENIX* GUI has been designed around individual programs, many of the most common automation strategies for phasing and model building are implemented in the wizards (Terwilliger *et al.*, 2006 ▶, 2008 ▶, 2009 ▶). The GUI provides a framework for further simplifying the process, primarily by linking related actions and easing the transition between programs. In the interface for *phenix.refine*, for instance, common tasks such as setting up restraints and adding hydrogen or deuterium atoms (*via* the *phenix.ready_set* program), identification of noncrystallographic symmetry (NCS) groups (*phenix.simple_ncs_from_pdb*), rapid identification of translation/libration/screw (TLS) groups (*phenix.find_tls*), and assigning secondary structure for the purpose of generating hydrogen-bond restraints (*phenix.secondary_structure_restraints*) are easily accessed and the results incorporated into the refinement input. At the end of each run, the validation suite is automatically executed and the results displayed with refinement statistics (Fig. 3 ▶).

A simpler, but extremely important, type of automation is for programs to suggest appropriate next steps after a job is complete, and provide an easy and direct transition to other programs. For example, the wizard GUIs display buttons for launching *phenix.refine* with input fields filled in with data calculated by the wizard, and for running other wizards where appropriate. This allows new ‘pipelines’ to be constructed by chaining of GUI modules instead of integrating programs at a lower level, such as molecular replacement single-wavelength anomalous diffraction (MR-SAD) phasing, which is performed in *PHENIX* simply by running the molecular replacement GUI, then clicking a button labeled ‘Run MR-SAD’ to launch a separate wizard with the required inputs. Future developments will address common parallel workflows, such as evaluating multiple molecular replacement search models, or validating several closely related structures simultaneously.

## 4. Conclusions and future directions

The combination of Python and the Phil configuration system has proved to be a highly productive environment for efficiently developing graphical interfaces, which greatly increase the accessibility of *PHENIX* to the broader community of structural biologists. New interfaces are added regularly, and because configurable parameters are now usually designed with GUI considerations in mind, their implementation typically requires only a small effort relative to the effort invested in developing the core algorithms. The integration with *Coot* and *PyMOL* provides a nearly seamless workflow for all aspects of structure determination and refinement starting from processed data, without requiring knowledge of the *PHENIX* command-line tools. This allows the training of researchers to focus on general principles and best practices, rather than advanced computer skills.

As development of *PHENIX* and the GUI continues, the underlying framework is constantly revised to improve modularity and enable more comprehensive unit testing. Lightweight applications using Phil can often be integrated into the *PHENIX* GUI without specialized Python code, and a growing set of utilities now uses this approach. Eventually the core GUI libraries will be extended to serve as a general-purpose framework for developing graphical tools built around the *cctbx*, incorporating related programs such as *LABELIT* (Sauter *et al.*, 2004 ▶).

## 5. Availability

The GUI is distributed as part of the *PHENIX* suite, which is freely available to academic users, both as binaries for standard Macintosh and Linux platforms and as source code. *PHENIX* can be obtained from http://www.phenix-online.org after online registration. The Phil implementation and many of the low-level graphical controls, toolkits and libraries are also available as part of the open-source *cctbx* project (http://cctbx.sourceforge.net).
